# Communication Problems After the Great East Japan Earthquake of 2011

**DOI:** 10.1017/dmp.2014.49

**Published:** 2014-06-13

**Authors:** Hitoshi Yamamura, Kazuhisa Kaneda, Yasumitsu Mizobata

**Affiliations:** Department of Critical Care Medicine, Osaka City University, Osaka, Japan

**Keywords:** communication, disasters, earthquakes, emergency medical service, communication systems

## Abstract

**Objectives:**

After the 2011 Great East Japan Earthquake, the resource utilization of and the problems encountered with communication devices were examined.

**Methods:**

A questionnaire survey was submitted to disaster medical assistance teams (DMATs) that were at the primary sites of destruction after the earthquake.

**Results:**

We collected data from 196 teams. During the first 4 days after the earthquake, the use of mobile phones, laptop computers, and landline phones was rated as poor to moderate, and satisfaction was very low, while satisfaction with satellite phones was rated as good to moderate (50%). The degree of satisfaction continued to increase gradually over time. Satellite phones, however, had several problems: poor reception, line instability, voice call use only, and inability to send large amounts of data.

**Conclusions:**

To ensure effective communication during the acute phase in the aftermath of large disasters, a new satellite communication device is needed that not only is portable, battery powered, and able to send large volumes of data, but also offers stable communication. (*Disaster Med Public Health Preparedness*. 2014:0:1–4)

The Great East Japan Earthquake occurred at 14:46 on March 11, 2011. It was catastrophic, registering 9.1 on the Richter scale. After the earthquake, a large tsunami occurred in the bay of the eastern Sea of Japan. This disaster brought damage to a wide area, and ongoing effects during the first 2 weeks post-disaster destroyed the local infrastructure.

After a disaster occurs, the communication infrastructure and its associated devices are crucial to the collection of information. Problems of inadequate information acquisition and exchange between the rescue staging care units and the control task force result in a confusion of tasks. Only a few reports of the communication status after the disaster have been published.^1^ ^,^ ^2^ Therefore, the objective of this study was to examine resource utilization of and problems encountered with communication devices when they were used in conjunction with medical treatment at sites immediately adjacent to the region affected by the 2011 Great East Japan Earthquake and tsunami.

## Methods

### Study Design

We conducted a questionnaire survey of disaster medical assistance teams (DMATs) that were at the primary sites of destruction following the Great East Japan Earthquake and resulting tsunami. In Japan, DMATs are defined as mobile medical teams that receive professional training. These teams of medical doctors, nurses, and logistics personnel are activated during the acute phase of a disaster.

We sent questionnaires to 375 randomly selected DMATs in Japan that provided medical support just after the earthquake disaster and performed medical services during the acute and subacute phases after the earthquake. A letter and the questionnaire were sent to hospitals with a DMAT on October 18, 2012, and the questionnaires were collected from October 18 to November 10, 2012. The questionnaire was sent to all areas in Japan, including those affected and unaffected by the earthquake. In this disaster, DMATs performed 2 types of medical service. The first was acute-phase services in the disaster such as triage, initial treatment, and patient transport, and the second was medical care in the affected area including public health service.

### Measures

From the questionnaires, we collected data that concerned the location and active period during which the DMATs provided medical support, the kinds of communication tools used, the transmission status of these devices, and the teams’ satisfaction with the communication situation.

Level of satisfaction with the communication situation was divided into 3 categories: good, moderate, and poor. We defined good as being able to use the communication tool without a problem; moderate as having some problem with the communication tool, although the problem was not great considering the usual condition of communication; and poor as having a serious problem with the communication tool.

## Results

### Sample Characteristics

Data from 196 teams were collected and could be evaluated. In the acute phase after the earthquake, the percentage of time the DMATs used each device as their communication tool was as follows: mobile phones, 30%; satellite phones, 23%; laptop computers, 21%; transceivers (radio-handy type), 15%; and landline phones, 11%.

During the first 2 days after the earthquake, 154 DMATs were working at staging care units in conjunction with the control task force and in hospitals within the affected area. During this period, the satisfaction with mobile phones was the worst except for 6 teams working in the cities of Sendai, Fukushima, and Mito. Satisfaction with satellite phones was similar between DMATs using these phones in affected and unaffected areas. At 3 days after the earthquake, medical teams were offering medical treatment and public health services in affected hospitals or disaster shelters such as schools, gymnasiums, and public halls in the affected area.

### Levels of Satisfaction With the Communication Devices

Mobile phones, laptop computers, and landline phones were often used at the staging care units or by the task force in unaffected areas within the first 4 days after the earthquake. During these first 4 days, mobile phone use was rated as poor to moderate, and satisfaction was very low (Figure). However, at 5 days after the earthquake, satisfaction with mobile phone use began to gradually increase. Satisfaction with laptop computer and landline phone use was similar to that with mobile phone use. In contrast, satisfaction with satellite phones was rated as good to moderate (at 50%) during the first 4 days after the earthquake, and this degree of satisfaction continued to increase gradually over time (Figure).

**FIGURE:**
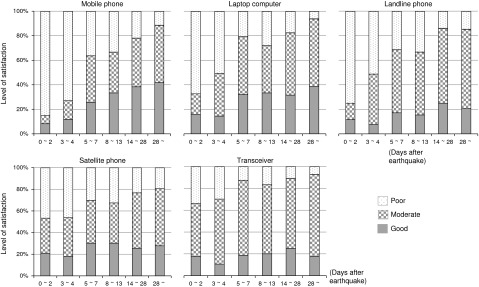
Levels of Satisfaction With Mobile, Landline, and Satellite Phones; Laptop Computers; and Transceivers in the First 28 Days After the Earthquake.

### Reasons for Poor Evaluation

The reasons for poor satisfaction with mobile phones, laptop computers, and landline phones were mostly due to disconnection from the network and poor reception. Poor operability affected the satisfaction with satellite phones; they could not be used to send large amounts of data, because they could only be used to communicate by voice.

### Communications Equipment Required in the Future

After the experience of the Great East Japan earthquake, the 196 responding DMATs indicated the following features were the most necessary in future communications equipment: easy to connect (187 responses), good battery life (162 responses), stability (156 responses), speed (123 responses), portability (121 responses), operability (95 responses), durability (92 responses), and the ability to transmit high volumes of data (65 responses).

## Discussion

The Great East Japan Earthquake resulted in profound damage and confusion to the communication system of the affected area. On the first day after the earthquake, discontinued service gradually increased and reached a peak 2 days after the earthquake on March 13.^3^ This phenomenon of gradually increasing loss of service was thought to be affected by the tsunami. However, by 7 days after the earthquake, line disconnections had been reduced to a low of 20%.

The number of mobile phone base stations without service peaked at 2 or 3 days after the earthquake and then gradually recovered.^3^ At 7 days after the earthquake, the total number of mobile phone base stations without service was less than 3000 stations. In addition, during the first and second days after the earthquake, connections for landline and mobile phones were limited.^3^

When a landline phone or mobile phone switchboard handles a large number of calls, traffic control equipment senses this condition and a computer limits the number of calls automatically. The control of landline connections was regulated in this manner, and limited the total number of connections to 10% to 20%. Mobile phone connections were handled similarly; these connections were limited to 5% to 30% of all connections. However, few limitations were placed on the sending of e-mails by mobile phone. Thus, the problem with the communication system on the first day of the disaster was that calls from landline and mobile phones were limited by as much as 80% to 90% of normal traffic.

Facilitation of communication within a disaster-affected area is an important problem, along with the ability to provide timely and relevant information and communication to the affected population from the unaffected area. In Japan, almost all hospitals and local authorities have priority landline phones for use in a disaster. In this survey, we could not distinguish whether a common or priority line was used for landline communications.

Also in Japan, it is required that the multichannel access (MCA) radio system be used for hospital-to-hospital or hospital-to-local authority communications. MCA is a useful communication system because it is not affected by the automatic congestion control system that affects the public telephone network. However, the DMATs could not use MCA for their activities in the field during the disaster, and the system has limits on call times and locations where it can be used.

In general, information derives from many sources such as mobile phones, computers using the Internet, landline phones, and radio and television.^4^ During the Great East Japan Earthquake, laptop computers were used by many entities such as the task force, hospitals, and disaster countermeasures office in locations affected by the disaster. Connections to the Internet in Japan are via asymmetric digital subscriber line (ADSL), optical fiber communications, cable Internet (connection), and wireless fidelity (Wi-Fi). In this survey, we could not identify the type of Internet connections used at each location that used computers.

Huge natural disasters, such as earthquakes, typhoons, tsunamis, and volcanic eruptions, usually cause a shutdown of the communications infrastructure. Many types of communications equipment are affected by the power failures and destruction of communication lines. In this incident, much information was derived from often incomplete and sketchy voice communications provided by radio transceivers, satellite phones, and disaster prevention radios. Consequently, this situation resulted in a partial understanding of the situation on the ground, which produced operational and tactical errors.

The development of a telecommunication capability that guarantees multimodal data transmission including voice, data, imagery, and videos in severely compromised conditions has become of paramount importance in the United States.^5^ ^–^ ^7^

Difficulties with post-disaster telecommunications are not restricted to wired and fixed networks but affect wireless networks just as intensely: after transmission through a short radio wave path, wireless communication systems eventually connect to a ground segment through an access point. Nevertheless, wireless technology is probably the only modality offering the potential for a relatively rapid post-disaster reconstruction of at least an embryonic version of a functional and capable telecommunications infrastructure.^5^ ^,^ ^8^ ^,^ ^9^

In Japan, portable satellite phones were useful due to narrowed roads and the varied topography in the disaster area. The earthquake and tsunami destroyed buildings and roads, leaving large piles of rubble blocking the roads and railways and creating a situation in which cars and trucks could not gain access to affected areas for rescue or to deliver relief supplies.

The findings of our survey showed that the portable satellite phone was found to be a useful communication tool during this disaster. During the immediate 4-day period after the disaster, service to many of the mobile base stations and landlines in the affected area was cut. In such conditions, satellite phones and transceivers become useful devices for communication.^10^

However, some problems were also experienced with the Japanese satellite phones after the earthquake. These problems included poor reception, line instability, and the fact that these phones could only be used for voice calls, which meant that large amounts of data could not be sent. Before the Great East Japan Earthquake, DMAT members were trained to operate with simple and small amounts of data. However, the responses from this survey identified a clear need for DMATs to be able to send a large amount of data during a large-scale disaster.

To lessen communication problems in future disasters, we identified the following needed resources. Exclusive communication lines are necessary for medical emergencies. Also, many DMATs thought that social networking services (SNS) through mobile phones were useful and effective in the early days after the earthquake; they used Twitter and Facebook to communicate during the disaster. If mobile phones can be used during a disaster, SNS may be one a very useful means of communication.

We also noted that a new satellite phone needs to be developed that connects easily to the communication network and Internet and provides good line stability. Finally, a system is needed whereby high volumes of data can be transmitted by satellite phone. Last year, a new battery-powered Japanese satellite phone was introduced that weighs only 1300 g with battery and can be used with the Internet. We expect that this phone will become a part of the communications equipment used in future disasters.

## Conclusions

In the acute phase after the Great East Japan Earthquake of 2011, medical teams mostly used mobile phones, laptop computers, and satellite phones to communicate. During the first 7 days after the disaster, the communications infrastructure was severely disabled, and it was difficult to use mobile and landline phones and computers with Internet service with the communication system. However, by the end of this initial 7-day period, these devices could be used in the affected area. The development of a new battery-powered satellite communication device that can transmit high volumes of data, is portable, and offers stable communication is necessary to ensure effective communications in the future during the acute phase of such large-scale disasters.

## Funding and Support

This work as supported by Japan Society for the Promotion of Science KAKENHI grant No. 24659799.
